# Characterization and Processing of Table Olives: A Special Issue

**DOI:** 10.3390/foods9101469

**Published:** 2020-10-15

**Authors:** Beatriz Gandul-Rojas, Lourdes Gallardo-Guerrero

**Affiliations:** Chemistry and Biochemistry of Pigments, Food Phytochemistry Department, Instituto de la Grasa (CSIC), Campus Universitario Pablo de Olavide, Edificio 46, Ctra. Utrera km 1, 41013 Sevilla, Spain; lgallardo@ig.csic.es

**Keywords:** functional food, bioaccessibility, microbiological quality, mineral nutrients, nutritional properties, predictive models, pigment composition, sensory analysis, starter cultures, user-friendly spreadsheet, volatile composition

## Abstract

Table olives are recognized as an essential component of the Mediterranean diet, having been explicitly included in the second level of its nutritional pyramid as an aperitif or culinary ingredient, with a recommended daily consumption of one to two portions (15–30 g). Producers demand innovative techniques improving the performance and industrial sustainability, as well as the development of new products that respond efficiently to increasingly demanding consumers. The purpose of this special issue was to publish high-quality papers with the aim to cover the state-of-the-art, recent progress and perspectives related to characterization and processing of table olives. Two reviews offer an overview about the processing and storage effects on the nutritional and sensory properties of table olives, as well as the main technologies used for olive fermentation, and the role of lactic acid bacteria and yeasts characterizing this niche during the fermentation. A total of 10 research papers cover a broad range of aspects such as characterization of their chemical composition, bioavailability, advances in the processing technology, chemical and microbiological changes, optimized use of starter cultures for the improvement of the different fermentative processes, and new strategies to reduce sodium and additives to stabilize the organoleptic properties and avoid defects.

The olive tree (*Olea europea* L.) is a widely distributed plant originating in the Mediterranean region. It is the most cultivated fruit tree in the world, surpassing 11 M ha. Although the olive fruits are mostly destined to obtain the highly valued olive oil, 11% of them are processed for direct consumption as table olives. This food of high nutritional value was sustenance and a source of calcium for the Mediterranean inhabitants in times of scarcity. Table olives are currently consumed as an appetizer and/or highly healthy culinary ingredients for their low sugars content, high monounsaturated fatty acids content, and additional contribution of fiber, minerals, vitamins, and bioactive components.

The olive fruit is a bitter drupe that has to be processed to transform it into an appetizing and edible food. There is a wide range of production styles, depending on the variety, ripening degree, and type of fruit (whole or split), aimed at hydrolyzing and/or diffusing to the brine the bitter oleuropein glucoside. The most widespread systems are those that use an alkaline hydrolysis or a slow acid and enzymatic hydrolysis. In addition, a fermentative process by lactic acid bacteria or yeasts is usually developed to increase palatability.

Producers demand innovative techniques improving the performance and industrial sustainability, as well as the development of new products that respond efficiently to increasingly demanding consumers. Foods with optimal nutritional characteristics, high quality, and safety, improved organoleptic characteristics, and with reduction of additives, are highly demanded. Under this framework, researchers were invited to participate in this special issue with original research papers or review articles focused on novel aspects related to table olives: characterization of their chemical composition, functional properties, and bioavailability of phytochemicals, as well as advances in the processing technology and waste treatment, including emerging techniques and optimized use of starter cultures for the improvement of the different fermentative processes. New strategies were also expected to reduce sodium and additives, to stabilize the organoleptic properties and avoid defects. Conservation methods aimed at extending the shelf life of highly valued artisanal products were also a goal. Likewise, analytical methods and prediction models for the traceability of the products and the detection of fraudulent practices related to the use of unauthorized additives were of interest. Fortunately, our proposal has had a good response, and a wide range of the above topics have been covered in this Special Issue thanks to 12 high-quality contributions.

Perpetuini et al. [1] present an overview of the main technologies used for olive fermentation and the role of lactic acid bacteria and yeasts characterizing this niche during the fermentation. The authors offer particular attention to the selection and use of microorganisms as starter cultures to speed up the process and improve the safety of table olives. In addition, they discuss the development and implementation of multifunctional starter cultures in order to obtain health-oriented table olives.

On the other hand, with the aim of giving an up-to-date overview of the processing and storage effects on the nutritional and sensory properties of table olives, Conte et al. [2] analyze the most relevant literature of the last twenty years in the review “Table Olives: An Overview on Effects of Processing on Nutritional and Sensory Quality”. According to this analysis [2], the nutritional properties of table olives are mainly influenced by the processing method used, even if preharvest-factors such as irrigation and fruit ripening stage may have a certain weight. Data reveal that the nutritional value of table olives depends mostly on the balanced profile of polyunsaturated and monounsaturated fatty acids and the contents of health-promoting phenolic compounds, which are best retained in natural table olives. Studies on the use of low-salt brines and of selected starter cultures have shown the possibility of producing table olives with an improved nutritional profile. Sensory characteristics are mostly process-dependent, and a relevant contribute is achieved by starters, not only for reducing the bitterness of fruits, but also for imparting new and typical taste to table olives. Findings reported in this review confirm that table olives surely constitute an important food source for their balanced nutritional profile and unique sensory characteristics.

In the work “Benefits of the Use of Lactic Acid Bacteria Starter in Green Cracked Cypriot Table Olives Fermentation”, Anagnostopoulos et al. [3] study the microbial and physicochemical changes of Cypriot green cracked table olives during spontaneous or controlled fermentation process at industrial scale. For this purpose, the authors processed Cypriot green cracked table olives directly in brine (natural olives), using three distinct methods: spontaneous fermentation, inoculation with commercial lactic acid bacteria at a 7%, or a 10% NaCl concentration. Sensory, physicochemical, and microbiological alterations were monitored at intervals, and major differences were detected across treatments. Results indicated that the predominant microorganisms in the inoculated treatments were lactic acid bacteria, while yeasts predominated in control. As a consequence, starter culture contributed to a crucial effect on olives fermentation, leading to faster acidification and lower pH, and inhibition of enterobacteria growth in a shorter period and at a significantly lower salt concentration, compared to the spontaneous fermentation. Likewise, the degradation of oleuropein was achieved faster in inoculated treatments, thus producing higher levels of hydroxytyrosol. Notably, the reduction of salt concentration, in combination with the use of starter, accented novel organoleptic characteristics in the final product, as confirmed from a sensory panel; hence, it becomes obvious that the production of Cypriot table olives at reduced NaCl levels is feasible.

The microbial starters used for table olives can be made by a few species and strains (selected starter cultures) or can consist of an indefinite number of microorganisms (natural biodiverse starter cultures). In order to select the best candidates to be used as starters, Paba et al. [4] carry out a comparative study between twenty-seven *Lactobacillus pentosus* strains, and the undefined starter for table olives from which they were isolated. Strains were characterized for their technological properties: tolerance to low temperature, high salt concentration, alkaline pH, and olive leaf extract; acidifying ability; oleuropein degradation; hydrogen peroxide and lactic acid production. Then, the authors selected two strains with appropriate technological properties, and they compared table olive fermentation in vats, with the original starter (autochthonous and undefined biodiverse starter, SIE), the selected double-strain starter (DSS), and without starter (natural fermentation, NF). Starters affected some texture profile parameters. The SIE resulted in the most effective Enterobacteriaceae reduction, acidification, and olive debittering, while the DSS batch showed the lowest antioxidant activity. Overall, the authors conclude that the best candidate strains cannot guarantee better fermentation performance than the undefined biodiverse mix from which they originate.

The presence of volatile organic compounds (VOCs) in table olives plays an unquestionable role in their sensory appeal. In the work “Lactic acid bacteria and yeast inocula modulate the volatile profile of Spanish-style green table olive fermentation”, Benitez-Cabello et al [5] designed a study to support that the VOCs profile of olive fermentation may be modulated by the addition of starter culture, as suggested by different researchers. For this purpose, the authors analyzed the VOCs in brines of Manzanilla Spanish-style green table olive fermentations that were inoculated with two strain of *Lactobacillus pentosus* (LPG1 and Lp13), one of *Lactobacillus plantarum* Lpl15, the yeast *Wickerhanomyces anomalus* Y12, and a mixed culture of all them, and they applied diverse multivariate statistical techniques for studying the results. After fermentation (65 days), a total of 131 volatile compounds were found, but only 71 showed statistical differences between, at least, two fermentation processes. Results showed that inoculation with Lactobacillus strains, especially *L. pentosus* Lp13, reduced the formation of volatile compounds. On the contrary, inoculation with *W. anomalus* Y12 increased their concentrations with respect to the spontaneous process, mainly of 1-butanol, 2-phenylethyl acetate, ethanol, and 2-methyl-1-butanol. Furthermore, biplot and biclustering analyses segregated fermentations inoculated with Lp13 and Y12 from the rest of the processes. The authors point out that the use of sequential lactic acid bacteria and yeasts inocula, or their mixture, in Spanish-style green table olive fermentation, could be advisable practice for producing differentiated and high-quality products with improved aromatic profile.

The topics related to the composition of volatiles and the sensory analysis of table olives have been also addressed by Sánchez-Rodríguez et al. [6] in HydroSOStainable table olives (HydroSOS), which are produced from olive trees grown under regulated deficit irrigation (RDI) strategies. In this contribution, the authors study the volatile composition, the sensory profile and the consumer opinion and willingness to pay (at three locations) for HydroSOS table olives (cv. Manzanilla), produced from three RDI treatments and a control, and processed as Spanish-style. Volatile composition was affected by RDI, by increasing alcohols, ketones, and phenolic compounds in some treatments, while others led to a decrease in esters and the content of organic acids. Descriptive sensory analysis (10 panelists) showed an increase in green-olive flavor with a decrease in bitterness in the HydroSOS samples. Consumers, after being informed about the HydroSOS concept, preferred HydroSOS table olives to the conventional samples and were willing to pay a higher price for them. Finally, green-olive flavor, hardness, crunchiness, bitterness, sweetness, and saltiness were defined as the attributes driving consumer acceptance of HydroSOS table olives.

There is vast experience in the application of sensory analysis to green Spanish-style olives, but black olives have received scarce attention and panelists have less experience on the evaluation of this presentation. In relation to this matter, Lopez-López et al. [7], contribute to this special issue with the work entitled “Panel and panelist performance in the sensory evaluation of black ripe olives from Spanish Manzanilla and Hojiblanca cvs.”. Using previously developed lexicon, ripe black olives from Manzanilla and Hojiblanca cultivars from different origins were sensorily analyzed according to the Quantitative Descriptive Analysis (QDA). The panel (eight men and six women) was trained, and the QDA tests were performed following similar recommendations as for green olives. The data were examined while using SensoMineR v.1.07, programmed in R, which provides a diversity of easy to interpret graphical outputs. The repeatability and reproducibility of panel and panelists were good for product characterization. However, the panel performance investigation was essential in detecting details of panel work (detection of panelists with low discriminant power, those that have interpreted the scale in a different way than the whole panel, the identification of panelists who required training in several/specific descriptors, or those with low discriminant power). Besides, the study identified the descriptors of hard evaluation (skin green, vinegar, bitterness, or natural fruity/floral).

Aspects related to chemical composition, functional properties, and bioavailability of phytochemicals in table olives have also been addressed in this special issue. In this frame, Lanza et al. [8] focused their research on the study of the influence of different brining processes with iodized and non-iodized salt on mineral content, microbial biodiversity, sensory evaluation, and color change of natural fermented table olives. Iodized salt has been used in food processing to prevent iodine deficiency disorders. Then, fresh olives of Carolea and Leucocarpa cvs. were immersed in different brines prepared with two different types of salt: the PGI “Sale marino di Trapani” and the same salt enriched with 0.006% of KIO_3_. PGI sea salt significantly enriches the olive flesh in macro-elements such as Na, K, and Mg, and microelements such as Fe, Mn, Cu, and Zn. Instead, Ca decreases, P remains constant, while iodine is present in trace amounts. In the olives fermented in iodized-PGI sea salt brine, the iodine content reached values of 109 µg/100 g (Carolea cv.) and 38 µg/100 g (Leucocarpa cv.). The relationships between the two varieties and the mineral composition were explained by principal component analysis (PCA) and cluster analysis (CA). Furthermore, analyzing the fermenting brines, iodine significantly reduces the microbial load, represented only by yeasts, both in Carolea cv. and in Leucocarpa cv. Candida is the most representative *genus*. The sensory and color properties weren’t significantly influenced by iodized brining. Only Carolea cv. showed significative difference for b* parameter and, consequently, for C value. The authors point out that knowledge of the effects of iodized and non-iodized brining on table olives will be useful for developing new functional foods, positively influencing the composition of food products.

The research conducted by López-López et al. [9] studies, for the first time, the bioaccessibility of the mineral nutrients in table olives darkened by oxidation (ripe olives) and their contributions to the recommended daily intake (RDI), according to digestion methods (Miller’s vs. Crews’ protocols), digestion type (standard vs. modified, standard plus a post-digest re-extraction), and mineralization system (wet vs. ashing). The digestion protocols had significant effects on the bioaccessibility estimation of ripe olive mineral nutrients. Overall, Miller’s protocol led to higher bioaccessibilities of Na, K, Ca, Mg, and Fe than the Crews’ method. The modified protocols improved most of the values, and they were useful to evaluate the strength of the linkage between some elements and olive flesh components. Monovalent minerals (Na and K) were hardly bound and completely bioaccessible. In contrast, the noticeable presence of divalent (and P) elements in the final solid residue indicated that at least some of them can still be strongly linked to olive flesh even after digestion. The modified Miller’s protocol, regardless of the mineralization system, led to the overall highest bioaccessibility values in ripe olives, which were: Na (96%), K (95%), Ca (20%), Mg (73%), Fe (45%), and P (60%). Their potential contributions to the RDI, based on these bioaccessibilities and 100 g olive flesh service size, were then 29, 0.5, 4, 3, 33, and 1%, respectively. This investigation has led to the proposition of the modified Miller’s protocol, which includes a post-digest re-extraction, for further studies on the bioaccessibility of mineral nutrients in table olives.

Alkaline treatment is a key stage in the production of green table olives and its main aim is rapid debittering of the fruit. However, its action is complex and structural changes in the olive, and loss of bioactive components, also occur. Because chlorophylls are one of the bioactive components seriously affected, Berlanga-Del Pozo et al. [10] designed a work aimed to investigate the effect of the alkaline treatment on these pigments that are responsible for the characteristic bright green color of table olives not preserved by fermentation. Specifically, the authors investigated the effect of nine combinations of two important parameters of the alkali treatment (NaOH concentration and treatment time) on green table olives processed in the *Campo Real style* and preserved for 1 year under refrigerated conditions. They found a direct relationship between the intensity of the alkali treatment and the degree of chlorophyll degradation, with losses of more than 60% being recorded when NaOH concentration of 4% or greater were used. Oxidation with opening of the isocyclic ring was the main structural change, followed by pheophytinization and degradation to colorless products. To a lesser extent, decarbomethoxylation and dephytylation reactions were detected. An increase in NaOH from 2% to 5% reduced the treatment time from 7 to 4 h, but fostered greater formation of allomerized derivatives, and caused a significant decrease in the chlorophyll content of the olives. However, NaOH concentrations between 6% and 10% did not lead to further time reductions, which remained at 3 h, nor to a significant increase in oxidized compounds, though the proportion of isochlorin *e4*-type derivatives was modified. Chlorophyll compounds of series *b* were more prone to oxidation and degradation reactions to colorless products than those of series *a*. However, the latter showed a higher degree of pheophytinization, and, exclusively, decarbomethoxylation and dephytylation reactions.

Another important aspect in table olive processing concerns advances in conservation methods aimed at extending the shelf life of highly valued artisanal products, maintaining microbial quality, and ensuring safety. The *Clostridium sp*. is a large group of spore-forming, facultative or strictly anaerobic, gram-positive bacteria that can produce food poisoning. The table olive industry is demanding alternative formulations to respond to market demand for the reduction of acidity and salt contents in final products, while maintaining the appearance of freshness of fruits. In the work by Valero et al. [11], the authors develop logistic regression models for non-adapted and acid-adapted *Clostridium sporogenes* strains to study the influence of pH, NaCl, and incubation time on the probability of germination of their spores. They select the factor ranges so that the model could be applied to table olive processing. A *Clostridium sporogenes* cocktail was not able to germinate at pH < 5.0, although the adaptation of the strains produced an increase in the probability of germination at 5.0–5.5 pH levels and 6% NaCl concentration. At acidic pH values (5.0), the adapted strains germinated after 10 days of incubation, while those that were non-adapted required 15 days. At pH 5.75 and with 4% NaCl, germination of the adapted strains took place before 7 days, while several replicates of the non-adapted strains did not germinate after 42 days of storage. The model was validated in natural green olive brines with good results (>81.7% correct prediction cases). The information will be useful for the industry and administration to assess the safety risk in the formulation of new processing conditions in table olives and other fermented vegetables. 

Finally, Bevilacqua et al. [12] also contributes to this section on microbial quality and safety assurance of the Special Issue. The purpose of their manuscript was to develop a decision support tool based on simple input parameters to assess the potential for spoilage of green olives processed by the *Spanish-style* during the post-fermentation stage. The duration of this stage is quite variable (from a month to a year) and depends on demand and market prices. If pH and NaCl are not strictly controlled, a microbial spoilage can occur due to a variety of microorganisms (*Aerobacter*, bacilli, propionibacteria, oxidative yeasts, molds, etc.). In this paper, the authors propose a user-friendly spreadsheet (Excel interface), a designated MoS (Micro-Olive-Spreadsheet), as a tool to point out spoiling phenomena in *Bella di Cerignola* olive brines. The spreadsheet was designed as a protected Excel worksheet, where users input values for the microbiological criteria and pH of brines, and the output is a visual code, much like a traffic light: three red cells indicate a spoiling event, while two red cells indicate the possibility of a spoiling event. The input values are: (a) Total Aerobic Count (TAC); (b) Lactic Acid Bacteria (LAB); (c) yeasts; (d) staphylococci; (e) pH. TAC, LAB, yeasts, and pH are the input values for the first section (quality), while staphylococci count is the input for the second section (technological history). The worksheet can be modified by adding other indices or by setting different breakpoints; however, it is a simple tool for an effective application of hazard analysis and predictive microbiology in table olive production.

To conclude, the present special issue consists of 10 original research papers and two review articles. The research papers, focused on recent research advances related to characterization and processing of table olives, have covered microbiological and chemical changes in table olives during spontaneous or controlled fermentation employing different cultivars [3], characterization of their composition of volatiles and the sensory profile [5,6,7], mineral composition [8] and bioavailability [9], changes in bioactive components (chlorophylls) by processing [10], optimized use of starter cultures for the improvement of the different fermentative processes [4,5], and new strategies to reduce sodium and additives, to stabilize the organoleptic properties and avoid defects [11,12]. 
